# In Reply: TP53 Alteration Status and Tumor Mutational Burden Score: Prevalence and Prognosis in Head and Neck Squamous Cell Carcinoma

**DOI:** 10.1093/oncolo/oyac088

**Published:** 2022-05-13

**Authors:** Kimberly M Burcher, Harper L Wilson, Elena Gavrila, Arianne Abreu, Ralph B D’Agostino, Wei Zhang, Mercedes Porosnicu

**Affiliations:** Department of Internal Medicine, Wake Forest Baptist Medical Center, Winston-Salem, NC, USA; Department of Anesthesiology & Perioperative Medicine, Medical University of South Carolina College of Medicine, Charleston, SC, USA; Wake Forest School of Medicine, Winston-Salem, Salem, VA, USA; Department of Internal Medicine, LewisGale Medical Center, Salem, VA, USA; Biostatistics and Data Science, Wake Forest School of Medicine, Winston-Salem, NC, USA; Cancer Biology, Wake Forest School of Medicine, Winston-Salem, NC, USA; Medical Oncology and Hematology, Wake Forest Baptist Medical Center, Winston-Salem, NC, USA

## Abstract

This Letter to the Editor responds to recent comments by Jiang et al. and joins the recommendation for further investigations into the role of *TP53*alteration status and TMB as predictors of response to immunotherapy and survival in head and neck squamous cell carcinoma.

Immunotherapy continues to revolutionize oncology, yet progress is hindered by the lack of biomarkers that reliably predict response. This deficiency is especially limiting in head and neck squamous cell cancer (HNSCC), where immunotherapy benefits 20% of patients or less. Biomarkers, including tumor mutational burden (TMB), PD-L1 expression, and *TP53* mutations, have been implicated as predictors of response and survival in immunotherapy, but review reveals mixed results.^1-5^

In our previous manuscript, we discussed the prevalence and implications of various gene alterations in HNSCC, and showed that not only was *TP53* the most prevalent (present in 73.3% of patients’ tDNA and/or ctDNA samples), but it also predicted poor survival.^6^ Jiang et al.^7^ referenced these results and analyzed the prevalence and prognostic implications of *TP53* alterations in a cohort of 1661 patients treated with immunotherapy (128 with HNSCC). They reported that 45.3% of HNSCC patients had alterations in *TP53* (likely lessened by limitation of analysis to tDNA alone, although not clarified in the letter) and that such alterations were associated with decreased survival. The study further correlated *TP53* alterations with high TMB, concluding that these results indicate the prognostic value of *TP53* alterations for immunotherapy in HNSCC. To address these findings, we analyzed TMB in our 75-patients study. Methods were similar to the original paper.^6^ Tumor mutational burden was categorized into low (0-5 mutations/megabase) and high (6+ mutations/megabase) groups.

Seventy-two patients had available TMB; 47% (*n* = 34) had high scores and 53% (*n* = 38) had low scores. Patients with high TMB had improved survival when measured from time of sample collection (*P* = .0379) and diagnosis (*P* = .0176) in univariate (Figure 1A, B) and multivariate analysis (*P* = .01). High TMB score as a continuous variable also predicted improved survival (*P* = .014). No significant association between altered *TP53* and TMB was identified. As a continuous variable, TMB score and *TP53* alterations (present vs absent) were not significantly correlated in tDNA (average 7.54 vs 7.42; *P* = .95), ctDNA (average 7.78 vs 7.21; *P* = .75), or tDNA and/or ctDNA (average 7.48 vs 7.61; *P* = .95; Figure 1C-E). Similarly, TMB score as a categorical variable (high vs low) did not correlate with *TP53* alterations in any DNA samples (*P* = .60; *P* = .65; and *P* = .78, respectively).

**Figure 1.**
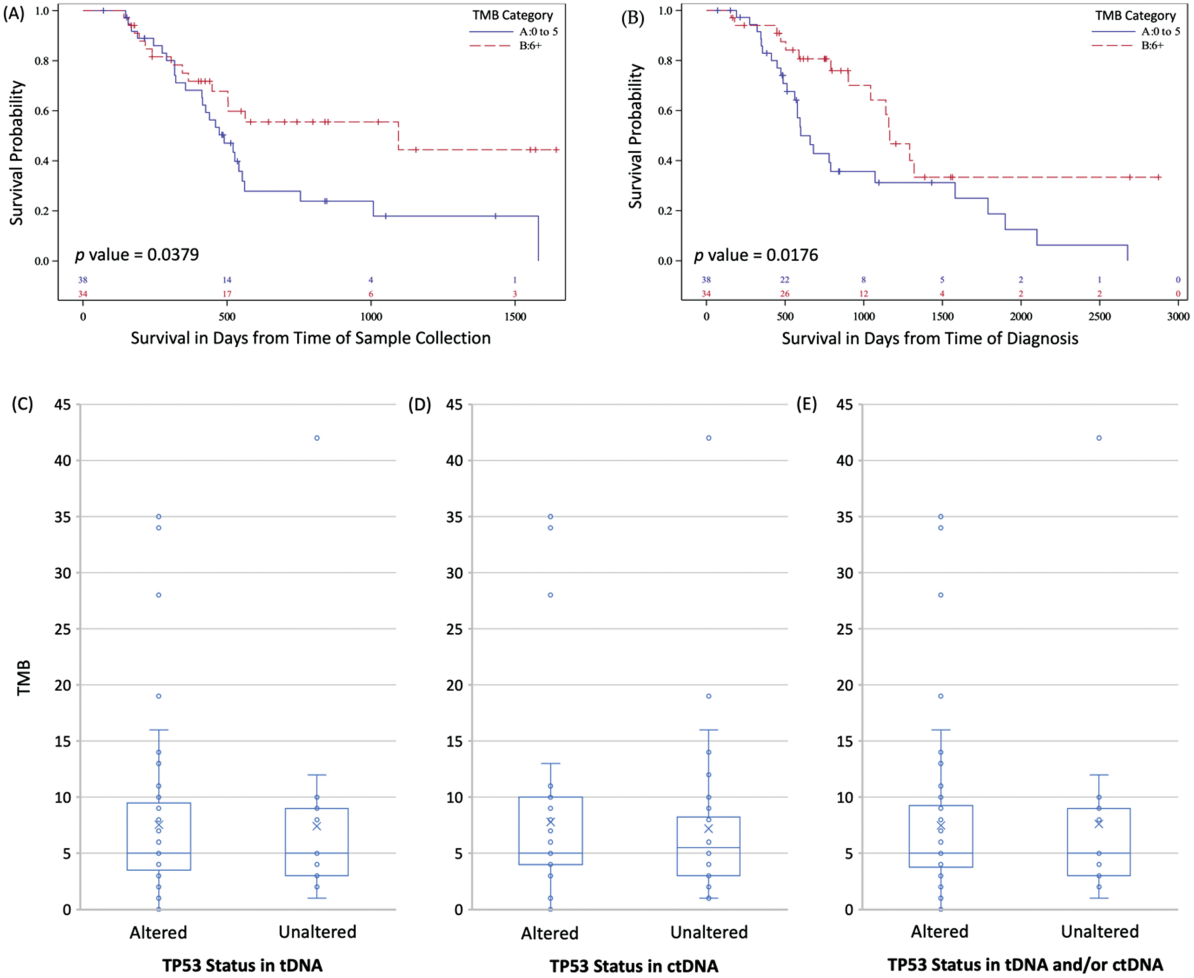
Kaplan-Meier analysis of survival regarding TMB (categorical) and distribution of TMB (continuous) across TP53 status. **(A**) Survival from time of sample collection in patients with TMB scores 0 to 5 vs 6 or above; **(B)** survival from time of diagnosis in patients with TMB scores 0 to 5 vs. 6 or above; **(C)** box plot charting TMB values (continuous) vs TP53 status in tDNA samples; **(D)** box plot charting TMB values (continuous) vs TP53 status in ctDNA samples; **(E)** box plot charting TMB values (continuous) vs TP53 status tDNA and/or ctDNA samples. Blue solid lines indicate survival curves for patients TMB 0 to 5; Red dashed lines indicate survival curves for patients with TMB 6 or above. In box plots, average TMB for each category are marked with an “X.” The median score is marked with a horizontal line within the box. Quartiles and outliers are depicted according to custom.

Only a small percentage of our study patients were treated with immunotherapy; therefore, analyses regarding immunotherapy response were not pursued. In the series by Jiang et al., all patients received immunotherapy, allowing correlative interpretation of the *TP53* status as potential treatment biomarker. The presence of *TP53* alterations was associated with decreased survival, and *TP53* alterations were associated with high TMB. These simultaneous findings make *TP53* alterations harder to interpret as prognosticator for immunotherapy. Nonetheless, there is literature to suggest the relationships between *TP53* status, TMB, survival, and immunotherapy response may be complicated by additional variables such as *TP53* protein functional status^8^ and the known association of *TP53* mutations with chromosomal aberations.^9^

The authors would like to thank Jiang et al for their interest and join the recommendation for further investigations into the role of *TP53* alteration status and TMB as predictors of response to immunotherapy and survival in HNSCC.
